# Oncology in Mozambique: Overview of the Diagnostic, Treatment, and Research Capacity

**DOI:** 10.3390/cancers15041163

**Published:** 2023-02-11

**Authors:** Satish Tulsidás, Filipa Fontes, Mariana Brandão, Nuno Lunet, Carla Carrilho

**Affiliations:** 1 Serviço de Oncologia Médica, Hospital Central de Maputo, nº 1653 Avenida Eduardo Mondlane, Maputo 1101, Mozambique; 2 EPIUnit–Instituto de Saúde Pública, Universidade do Porto, Rua das Taipas, n° 135, 4050-600 Porto, Portugal; 3 Laboratório para a Investigação Integrativa e Translacional em Saúde Populacional (ITR), Universidade do Porto, Rua das Taipas, n° 135, 4050-600 Porto, Portugal; 4 Departamento de Ciências da Saúde Pública e Forenses e Educação Médica, Faculdade de Medicina da Universidade do Porto, Rua Doutor Plácido da Costa, 4200-450 Porto, Portugal; 5 Unidade de Investigação em Enfermagem Oncológica, Centro de Investigação do Instituto Português de Oncologia do Porto, Rua Dr António Bernardino de Almeida, 4200-072 Porto, Portugal; 6 Department of Medical Oncology, Institut Jules Bordet, Université Libre de Bruxelles, Rue Meylemeersch 90, 1070 Anderlecht, Belgium; 7 Departamento de Patologia, Faculdade de Medicina, Universidade Eduardo Mondlane, Avenida Salvador Allende, nº 702, Maputo 1101, Mozambique; 8 Serviço de Anatomia Patológica, Hospital Central de Maputo, Avenida Eduardo Mondlane, nº 1653, Maputo 1101, Mozambique

**Keywords:** Mozambique, cancer care facilities, cancer screening, cancer vaccines, developing countries, neoplasm, registry

## Abstract

**Simple Summary:**

Mozambique is one of the poorest countries in the world. Because of the limited number of human and material resources, priority is usually given to infectious diseases because they are the main cause of death in Mozambique. However, with the increasing adoption of an unhealthy lifestyle, the number of deaths attributed to cancer is also increasing. This review summarizes cancer incidence and mortality, cancer prevention and screening, cancer care resources, and trends in cancer training and research in Mozambique, providing a background for the development of cancer care policies in the country.

**Abstract:**

Mozambique is one of the poorest countries worldwide, with nearly two thirds of the population living below the poverty line. Similarly to other less developed countries, there is a weak provision of health care for non-communicable diseases due to competing priorities with infectious diseases. Although the leading causes of death in Mozambique in 2019 were Acquired Immune Deficiency Syndrome/Human Immunodeficiency Virus and other sexually transmitted diseases and respiratory infections and tuberculosis, with increasing urbanization and westernization of lifestyles, deaths attributed to cancer are also on the rise. This review summarizes cancer burden, cancer prevention and screening, cancer care resources, and trends in cancer training and research in Mozambique, providing a background for the development of cancer care policies in the country.

## 1. Introduction

According to the World Health Organization (WHO), non-communicable diseases (NCDs) account for seven out of every ten deaths worldwide, of which 80% are due to cancers, cardiovascular diseases, chronic respiratory diseases, and diabetes; more than three quarters of all NCDs deaths occur in low- and middle-income countries [1].

In Africa, 1,109,209 new cancer cases (5.7% of the overall number in the world) and 711,429 deaths (7.1%) were estimated to have occurred in 2020 [2], and there is a projected increase of about 89.1% in the number of incident cases by 2040 [3]. The population aging and growth, as well as the increased prevalence of risk factors associated with socioeconomic development, such as tobacco and alcohol consumption, being overweight/obesity, physical inactivity, and changing reproductive behaviors [4,5], are among the main reasons that may explain the increasing burden of cancer in this context. In addition, there is a high prevalence of infections related to cancer [6]. Some improvements in access to cancer diagnosis and efforts for cancer registration may have also contributed to a growing number of known incident cases and cancer deaths.

Despite the increasing burden, low priority is usually given to cancer prevention and control in Africa, particularly in Sub-Saharan Africa (SSA), largely because other conditions, such as Human Immunodeficiency Virus (HIV) infection/Acquired Immune Deficiency Syndrome (AIDS), malaria, and tuberculosis, are more immediately perceived as threats to public health and compete for the limited available resources [7]. The lack of human resources, absence or limited access to screening programs, inadequate diagnosis capacity, poor access to treatment, lack of adequate infrastructure, low quality of cancer data, and lack of awareness among policymakers regarding the magnitude of cancer burden and its economic impact challenge the efforts towards cancer control in SSA [8,9,10].

Mozambique is one of the poorest countries worldwide, with a gross domestic product of 500 USD per capita in 2021 and nearly two thirds of the population living below the poverty line in 2014 [11]. In 2020, the estimated population was 31 million, the life expectancy at birth was 61 years, and approximately 45% of the population was under 15 years old [11]. The national health system covers less than half of the population, and the density of health professionals is low: 8.4 physicians, 33.4 nurses, and 760 hospital beds per 100,000 inhabitants [12]. Similarly to other less developed countries, there is a weak provision of health care for NCDs due to competing priorities with infectious diseases [13]. In fact, the leading causes of death in Mozambique in 2019 were HIV/AIDS and other sexually transmitted diseases and respiratory infections and tuberculosis [14]. However, with the urbanization and westernization of lifestyles, NCDs are also on the rise; from 1990 to 2019, cardiovascular diseases moved from the sixth to the fourth and cancer from the tenth to the sixth leading cause of death among Mozambicans [14].

Therefore, we aim to describe the cancer burden, cancer prevention and screening, cancer care resources, and trends in cancer training and research in Mozambique to provide a background for the development of cancer care policies in the country.

## 2. Cancer Epidemiology in Mozambique

There is no national population-based cancer registry in Mozambique. Local data on cancer patterns are scarce and have been limited to laboratorial [15], hospital-based [16,17,18,19,20], and two population-based registries [21]. This is similar to what has been observed in other Sub-Saharan African Countries, where population-based registries are not available at all or, when they exist, often cover only selected areas, mainly urban, or do not produce high-quality data [22].

The first cancer registration effort in Mozambique was made in 1956–1961 with a “cancer survey” of the population of Lourenço Marques (now Maputo city), conducted by the Pathology Department of the Miguel Bombarda Hospital (now Maputo Central Hospital (MCH)) [23].

The cases diagnosed at the Pathology Department of MCH between 1991 and 2008 were reported in 2015 [15]. In 2005, a population-based registry started in the city of Beira, in the center of the country [24]. In July 2014, the MCH implemented a hospital-based registry [20], and at the same time the population registry of the city of Maputo was also implemented [21]; the MCH cancer registry was estimated to contribute to almost 90% of the cases in the population-based registry.

The most recent local data shows cancer incidence estimates for Maputo city (2015–2017) of 105.1 cases per 100,000 men and 126.5 cases per 100,000 women; among men, the most frequent cancers were Kaposi sarcoma and prostate cancer, responsible for 26.7% and 15.9%, of cases, respectively, whereas in women, cervical cancer was by far the most common (29.9% of cases), followed by breast cancer (11.8% of cases) [21]. In Beira (2014–2017), the most frequent cancers were Kaposi sarcoma (33.9% of cases) and prostate cancer (7.8%) in men and cervical cancer (37.3%) and Kaposi sarcoma (10.8%) in women [21].

One study of cancer patterns from 1956–1961 to 2015–2016 showed a downward trend of infection-related cancers, such as liver and bladder cancers, as well as an emerging of cancers associated with the economic transition, such as prostate and breast cancers and cancers associated with HIV/AIDS [20].

According to GLOBOCAN, 25,446 new cases and 18,014 deaths were estimated to have occurred in Mozambique in 2020, corresponding to an incidence rate of 81.4 and a mortality rate of 57.6 per 100,000 [2].

Efforts have been recently made to report survival data from Mozambique (Table 1). Specifically, data from the population registry of the city of Maputo were used to estimate the survival rates of breast, colorectal, and cervical cancer patients diagnosed between 2014 and 2015 [25,26,27], though the estimates were based on a small number of cases and lacked precision. In addition, the survival rates were also available for patients diagnosed with incident breast (2015–2017) and esophageal (2012–2016) cancers that were followed at the MCH [28,29].

In the future, the national coverage and data quality of population-based registries need to be improved. In the same way, hospital-based registries will remain an important source of data for the evaluation of cancer management in this setting, and, therefore, efforts are also needed to increase their ability to assess cancer management in each institution.

## 3. Cancer Prevention

The incidence of cancer could be reduced by vaccination and decreasing exposure to risk factors.

Regarding vaccination, the Hepatitis B Virus (HBV) vaccine, for the prevention of hepatocellular carcinoma, was introduced into the National Immunization Program in 2001 [30,31]. The most recent immunization schedule comprises a three-dose HBV vaccination at 8, 12, and 16 weeks of age and a plan to introduce, by 2024, an HBV dose at birth to prevent vertical transmission [30]. According to the official data reported to WHO, Mozambique achieved a three-dose HBV national coverage of 79% in 2020, which remains below the established goal of a global coverage of 90% by 2020 [32]. Despite the growing trend in coverage, there are still many children who do not complete the vaccines as scheduled, as a result of dropouts between the first and the third doses [30].

The HPV vaccination, for the prevention of cervical cancer, was introduced into the National Immunization Program in November 2021 [33]. Before that, a pilot project supported by the Global Alliance for Vaccine Initiative (GAVI) and the Mozambican government was implemented in 2014/2015 targeting 10-year-old girls from the elementary schools of three cities covering the south, center, and north regions [31]. A study performed prior to the pilot project, including interviews with 1147 girls aged 10–19 years old, showed that 84% had heard of cervical cancer and 76% believed that cervical cancer can be prevented [34]. Additionally, 91% were willing to accept HPV vaccination, even if only one third recognized having heard about HPV infection [34]; awareness was higher in regions where the HPV vaccine pilot program had been conducted, probably reflecting the ongoing education and communication activities already occurring prior to the vaccination campaign, highlighting the critical role of health education in communities to improve adherence to vaccination.

In addition to vaccination, some other strategies have been implemented to reduce the impact of both infections related to cancer, such as HIV, and changes in lifestyle, including tobacco and alcohol consumption.

In 2003, the Ministry of Health introduced antiretroviral treatment (ART) services for adults (≥15-year old people) and children (0–14 years old) and in 2013 started universal access to antiretrovirals for all children under five years of age infected with HIV. In 2020, the estimated prevalence of HIV infection in Mozambique was 11.5% in the age group of 15 to 49 years; among those living with HIV, 81% know their status, and only 68% and 55% were on ART and lived with suppressed viral loads in 2020, respectively, which remains below the 90–90–90 target (90% of all those living with HIV know their status, 90% of those who know their HIV-positive status are on ART, and 90% of those on ART are virally suppressed) for 2020 [35].

Regarding tobacco consumption, data from the 2013–2018 Global Tobacco Survey yielded an overall prevalence of current use of any tobacco product among Mozambican adolescents (11–17 years) of 20.7% [36]. Among those aged 15–64 years, data from the 2005 WHO stepwise approach to surveillance (STEPS) Noncommunicable Disease Risk Factor Survey showed an overall prevalence of current consumption of any type of tobacco of 17.5% in women and 40.6% in men [37]. The WHO Framework Convention on Tobacco Control (FCTC), an international legal agreement with 182 parties covering more than 90% of the world population, which includes taxation measures, smoke-free environments, advertising bans, graphic warning labels, and age restrictions, was ratified by the Mozambican government only in 2017 [38], but some tobacco control policies were implemented in the country before that. For instance, tobacco prices increased by 85% in nominal terms in 2013–2016, reducing tobacco’s affordability [39]. Yet, the important contribution of tobacco to the total export value of agricultural commodities in Mozambique is one of the main obstacles addressed by local tobacco control policies [40]. According to the last global progress report on the FCTC’s implementation, Mozambique reported measures to achieve almost all the articles of the FCTC, except for article 5.3 that recognizes the protection of public health policies from tobacco industry interference [38].

Concerning alcohol consumption, the most recent national published data, based on the 2005 STEPS in Mozambique, showed that more than one quarter of women and more than half of men were current drinkers; 40% of the current drinkers reported to have had at least one binge-drinking occasion in the previous week [41]. The prevalence of current drinking increased with age and education among women and with income among men [41]. The Regulation on Control of Production, Marketing and Consumption of Alcoholic Beverages was approved in 2013, including measures such as the prohibition of selling alcoholic beverages to persons under 18 years of age, to those with signs of a mental disorder or intoxication, in fuel supply stations and respective convenience stores, and in schools or in the vicinity of educational establishments [42].

Regardless of the favorable measures for cancer prevention in the last two decades, further progress must be made to improve vaccination coverage and minimize the impact of economic globalization on the pattern of exposure to cancer risk factors in Mozambique.

## 4. Cancer Screening

Following the priorities defined in the National Strategic Plan for the Prevention and Control of Non-Communicable Diseases (2008–2014) [43], the National Program for the Prevention and Control of Cervical and Breast Cancer was launched in 2009 [44,45].

In 2009, a cervical cancer screening program targeting sexually active women aged between 30 and 55 years was introduced in four provinces—Maputo, Sofala, Nampula, and Zambézia—using assistance from Friends in Global Health, a partner supported by the Centers for Disease Control and Prevention and affiliated with the Vanderbilt Institute for Global Health that provided financial and training support for screening, including equipment purchases, facility renovations, and training health professionals [46]. The screening, which was gradually rolled out to all Mozambique provinces [47], uses the “screen-and-treat approach” based on visual inspection with the use of acetic acid followed by immediate treatment with cryotherapy [48], which has been proved to be an effective and low-cost alternative to cytology-based screening in low-income countries [49]. According to the National Program for the Prevention and Control of Cervical Cancer, women must be invited for screening when waiting for other services in primary health care units or in the context of health education campaigns on cervical cancer and screening performed in some communities [45].

In 2010, a study conducted in rural communities of the province of Zambézia showed that the willingness to be screened was high (84%), mainly among women who had previously heard about cervical cancer and about the screening program [50]. Yet, more recent national data based on the 2014/2015 WHO STEPS showed a very low screening uptake at the national level (3.0% of all women and 3.4% of women aged 30–55 years) [51]. In addition, the use of screening differs across regions. Among the target population, screening uptake was lower in the north (1.3%), higher in the south (5.8%), and 14.3% in Maputo city [51]. The low education level and the lack of knowledge about cervical cancer screening are some of the barriers to the screening program’s coverage [50,51,52]. The fact that the screening program only reaches those who seek health care and the limited proportion of health facilities that have implemented the program (44% of the total health care units in 2018) [47] preclude a higher impact of cervical cancer screening.

Regarding breast cancer screening, since 2008, there has been a recommendation for the early detection of breast cancer by breast self-examination and clinical examination by health professionals in primary health care units, mainly in family planning consultations [43], for women aged 30 years or more [44]. Due to financial constraints and the lack of qualified healthcare professionals, breast cancer screening by mammography is not recommended in the National Program for the Prevention and Control of Breast Cancer in Mozambique [53]. Additionally, to our knowledge, there is no published evidence regarding screening uptake and coverage.

Although prostate cancer was defined as a priority disease, along with cervical and breast cancers, in the Strategic National Plan for the Prevention and Control of Non-Communicable Diseases issued in 2008 [43] and in the National Plan for Cancer Control for 2019–2029 [54], so far there is not a program for prostate cancer screening in Mozambique.

The introduction in the medium term of HPV testing as a national primary screening method and in the short term of thermal ablation at the national level for treating intraepithelial cervical lesions as an alternative to cryotherapy could improve efforts for effective cervical cancer screening. Studies concerning cost-effective point-of-care screening tests and thermal ablation must be considered.

## 5. Cancer Diagnosis

### 5.1. Oncology Centers

The Mozambique National Health System is structured into four levels of care: primary, secondary, tertiary, and quaternary. The primary level is delivered through rural health centers, the secondary level consists of rural hospitals, the tertiary of provincial hospitals, and the quaternary of so-called central hospitals. The latter provides the most specialized care in the country and includes the MCH, the Beira Central Hospital (BCH), the Nampula Central Hospital (NCH), and the Quelimane Central Hospital (QCH) [55]. Although it is expected that these hospitals cover all medical specialties, including medical oncology, the latter is not yet available in the QCH [54]. Furthermore, MCH is the only hospital equipped with an oncological ward [56].

The availability of private health care services has been gradually increasing [54], although published data regarding their coverage and the services provided are lacking.

### 5.2. Pathology Services

Similarly to most of the Sub-Saharan African countries, Mozambique lacks the essential resources to implement effective cancer diagnosis [57,58].

Until 2016, the country had only three Anatomical Pathology Departments, namely, at MCH, BCH, and NCH in the south, center, and north of the country, respectively. In 2017, a new Pathology Department was created in the QCH, in the central region of Mozambique [59]. More recently, cytology units were created in some provincial and general hospitals in the country, and a basic pathology service was introduced in the Maputo periphery, namely, at the Mavalane General Hospital [59].

A survey conducted from October to December 2018 across the laboratories of the 4 central hospitals in Mozambique revealed that there were 14 pathologists (9 in Maputo, 2 in Beira, 2 in Nampula, and 1 in Quelimane) and 24 pathology/laboratory technicians (11 in Maputo, 5 in Beira, 6 in Nampula, and 2 in Quelimane) [55]. In addition to performing a greater number of routine diagnostic activities than the other central laboratories, such as autopsies, surgical pathology, exfoliative cervicovaginal cytology, and fine-needle aspiration cytology (FNAC), the MCH laboratory also generally received samples collected at facilities other than the four central laboratories [55].

Moreover, immunohistochemistry is only routinely available at the MCH, although the high cost of supplies and reagents limits its use [55], which compromises, for instance, the assessment of hormone receptors in breast cancer [60]. This is in accordance with the findings from a survey conducted in Africa showing that immunohistochemistry was available in approximately half of the 30 countries reporting data [57].

### 5.3. Imaging Resources

According to the WHO, in 2020, there were 1.6 mammographs, 1.6 computerized tomography (CT) scanners, and 0.4 magnetic resonance imaging (MRI) scanners per 10,000 cancer patients in Mozambique, which is below the median values of the WHO Africa Region (10.4 mammographs, 11.5 CT scanners, and 1.3 MRI scanners per 10,000 cancer patients). Positron emission tomography scanners are not yet available in Mozambique [61]. In addition to the scarcity of specialized imaging resources, some of the equipment available is obsolete or inoperative due to a lack of adequate maintenance [62].

In the future, efforts are needed for the improvement of the infrastructure of existing services, the maintenance of equipment, and the regular delivery of consumables and reagents. Increasing and centralizing immunohistochemistry in a center that is already doing this can save costs. Training technicians and residents on best practices in pathology and imageology and using standard procedures for reports must be reinforced in the short term. In addition, increasing communication with clinicians through participation in multidisciplinary groups and the introduction of telepathology and imageology between existing services in the country and between external consults will improve the quality of work and reduce the turnaround time of diagnosis.

## 6. Cancer Treatment

### 6.1. Surgical Oncology

The last report from the WHO on health human resources worldwide showed that there were 72.6 surgeons per 10,000 cancer patients in Mozambique in 2013; the corresponding median value in the WHO Africa Region was 52 per 10,000 in 2019 [61]. An article discussing cancer surveillance and resources in Mozambique stated that there are no oncological surgeons in this setting [24]. In Mozambique, like in many other developing countries, surgery is highly centered on acute conditions, and surgical treatment of cancer is performed by general surgeons with limited expertise in cancer surgery according to good oncological practices [63].

In a study performed in the MCH, cancer-specific educational surgical programs at different levels (namely, pre-graduate and continuing medical education) and the mobilization of adequate resources were implemented as needed to ensure the provision of adequate surgical oncological treatments [64]. Furthermore, regarding the latter, despite different activities being performed involving, for instance, Brazilian hospitals, the Calouste Gulbenkian Foundation from Portugal, and the MD Anderson Cancer Center from the USA to train Mozambique oncologists and oncology surgeons, there were no substantial changes in the practice of surgical oncology at MCH [56]. Recently, efforts have been made to define the content and the design of a fellowship curriculum (24 months) for practicing surgeons in surgical oncology that is suitable to be implemented in this context [56].

### 6.2. Systemic Treatment

According to the National Plan for Cancer Control (2019–2029), chemotherapy and hormonal therapy are available at the quaternary level, although their irregular supply leads to periods when some of the drugs are not available, which frequently causes non-compliance with the treatment or changes to the recommended treatment regimens [54]. Commonly prescribed drugs such as cisplatin and 5-fluorouracil (used as a first-line treatment for cervical carcinoma in Mozambique) or doxorubicin, cyclophosphamide, and paclitaxel (used for the treatment of breast cancer) are sometimes lacking in the public system. The same applies to hormonal treatments used in breast and prostate cancer, such as tamoxifen and goserelin. Imatinib, rituximab, and trastuzumab are available (with the same constraints described before), but aromatase inhibitors are not provided by the public system.

Regarding medical staff, there are only seven medical oncologists in Mozambique [24], three of whom are working at the MCH [64]. According to WHO data, there were 0.4 medical doctors per 10,000 cancer patients in Mozambique in 2019; the corresponding median value for the WHO Africa Region was 2 per 10,000 in 2019 [61].

### 6.3. Radiotherapy

Considering that prostate, cervical, and breast cancers are among the most common types of cancer in Mozambique, radiotherapy may play an important role as an adjuvant and/or palliative therapy in this setting.

Mozambique had a service for radiotherapy, based on cobalt, that was installed in the 1960s and worked until 1996. With time, the machine became obsolete and was discontinued, and there was also a lack of maintenance. After that, patients needing radiotherapy were referred to other countries, like South Africa, India, and Portugal.

The process of creating a new radiotherapy unit was started in 2006 with the adhesion of Mozambique to the International Atomic Energy Agency (IAEA), the creation of a National Authority for Atomic Energy (ANEA), and the development of training programs for radio-oncologists, medical physicists, radiotherapy technicians, and nurses. The radiotherapy unit started working in the MCH in August 2019. To our knowledge, there are no studies evaluating the impact of the new radiotherapy unit on patients’ outcomes.

Concerning human resources, there were three radiation oncologists in Mozambique in 2017, all of them working in the MCH [64]. In 2019, according to the WHO data, there were 1.2 radiation oncologists per 10,000 cancer patients in Mozambique, which is more than the median of the WHO African Region (0.0 radiation oncologists per 10,000 cancer patients) [61].

### 6.4. Multidisciplinary Care

Multidisciplinary tumor boards (MTBs) for breast cancer were introduced in 2016 in the MCH [28]. The breast cancer tumor board meeting takes place on a weekly basis, with the participation of surgeons, medical oncologists, pathologists, radiologists, and radiation oncologists. In addition to having contributed to a significant reduction in mortality among patients with early breast cancer, the implementation of the MTBs was demonstrated to be a cost-effective intervention [28]. Since 2016, although MTBs were also implemented for other cancers, namely, gynecological, head and neck, esophageal, lung, colorectal, and pediatric cancers, until now, there are no published data regarding the impact of these specific MTBs on cancer outcomes.

### 6.5. Palliative Care

Although the importance of palliative care has been recognized in the last National Plan for Cancer Control [54], it is still limited and not fully integrated into the health care system [65,66]. In addition, a recent study evaluating the general knowledge, attitudes, and practices of Mozambican physicians on palliative care concluded that there are also important knowledge gaps about palliative care among healthcare professionals [65].

Nevertheless, Mozambique is one of the six African countries, along with Rwanda, Swaziland, Tanzania, Zimbabwe, and Malawi, that provides a standalone palliative care service that is based in the Xai-Xai Provincial Hospital in the Gaza province [65,66]. Additionally, in the last two decades, some efforts have been made to increase the availability of pain units across the country. For instance, in 2001, a pain consultation started at the MCH, and in 2007, this was redefined as a pain unit, an independent service with its own physical space and multidisciplinary team; in 2019, a Palliative Care Service opened at the Pain Unit in response to the progressive increase in the number of patients with cancer admitted to the Pain Unit who needed relief from suffering [67]. Most activities performed were consultations (including psychological appointments), telephone assistance, home-based care, and intrahospital palliative care support. In 2019, approximately 40% of the palliative care patients were cancer patients [67].

A study performed between August 2018 and January 2019 at the MCH, NCH, BCH, and Xai-Xai Provincial Hospital revealed that 84% of the cancer patients had pain, most of them moderate to severe, which emphasizes the inadequate analgesia and the importance of qualified health professionals in pain management in this context [68].

Despite the difficulties presented regarding cancer treatment, efforts are being made following the strategies defined in the National Plan for Cancer Control (2019–2029), such as increasing cancer treatment capacity, expanding infrastructures, improving the organization of cancer treatment, and ensuring the availability of medicines, supplies, devices, and equipment for this purpose, with a view to ensuring standardization and quality in the treatments offered to patients.

## 7. Training and Mentoring Health Professionals

Recently, training and mentoring for health professionals involved in cancer care are being implemented in Mozambique through institutional partnership programs with Portugal, the USA, and Brazil, aiming to improve the diagnosis and treatment of oncological diseases [69,70].

Since 2014, the Calouste Gulbenkian Foundation and its partners have been providing support to medical and non-medical staff to be trained in Portugal in various cancer specialties during short periods of time [70]. This has allowed, for instance, the implementation of new cancer treatment techniques and multidisciplinary tumor boards in Mozambique [28,70].

In 2014, the Extension for Community Healthcare Outcomes (ECHO) Project, a low-cost teleconsulting and telementoring partnership with the MD Anderson Cancer Center with the collaboration of Brazilian institutions and Mozambican providers, started to be implemented in Mozambique [69]. This project aimed to increase local capacity and management skills toward the prevention of cervical cancer and the creation of a team of oncological gynecologists [71]. This approach, based on monthly videoconferences in Portuguese, was complemented by hands-on training at MCH and in Brazilian institutions [69].

Despite the importance of these programs, a strategy for the sustainability of all these initiatives needs to be considered.

## 8. Cancer Research

**Figure 1:**
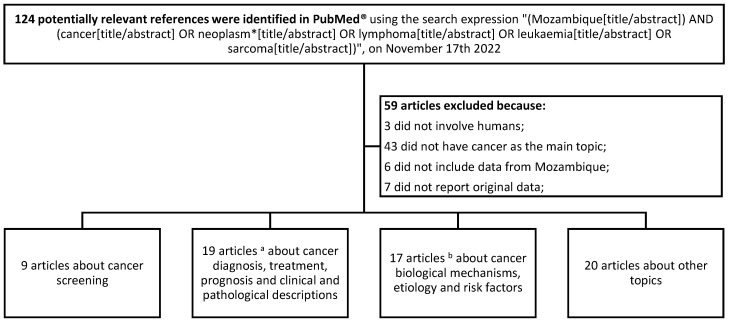
Flow chart describing the process of selection of articles with Mozambique cancer data from those published in PubMed^®^ between 1990 and 2022. ^a^ Includes articles about Kaposi sarcoma (N = 5); breast (N = 4), cervical (N = 2), esophageal (N = 2), liver (N = 1), bladder (N = 1), colorectal (N = 1), and oropharyngeal (N = 1) cancers; lymphoma (N = 1); and several cancers (N = 1). ^b^ Includes articles about cervical cancer (N = 5); Kaposi sarcoma (N = 3); liver (N = 1), breast (N = 1), esophageal (N = 1), stomach (N = 1), lung (N = 1), penile (N = 1), vulvar (N = 1), and eye (N = 1) cancers; and several cancers (N = 1). *: synonyms.

**Figure 2:**
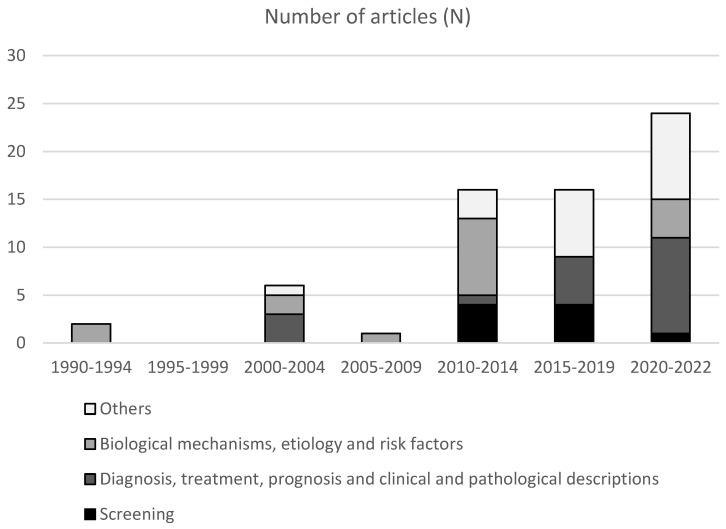
Number of articles published in PubMed^®^ from 1990 to 2022, according to the main topic Data were reported in periods of five years, except for the last category (2020–2022) where a period of three years was used.

Since 1990, at least 65 articles have been published in indexed peer-review journals with Mozambican cancer data (Figure 1). A total of nine (13.8%) have addressed cervical cancer screening; 19 (29.2%) diagnosis, treatment, prognosis, or clinical and pathological descriptions; 17 (26.2%) biological mechanisms, etiologic, or risk factors; and 20 (30.8%) other topics. The latter include mainly articles describing the experience with the implementation of training courses in oncology or describing temporal patterns in cancer incidence. Almost two thirds of the articles were published during the last eight years (Figure 2).

In a review of randomized, controlled trials on cancer registered on clinicaltrials.gov in November 2022 (N = 93,392 trials), only one took place in Mozambique (ClinicalTrials.gov Identifier: NCT05372484); nevertheless, from the 51,271 trials on cardiovascular disease worldwide, eight had already had the participation of Mozambican centers [72]. The lack of expertise and infrastructure capacity to conduct clinical trials and for data management, as well as the high cost of this type of research, have been pointed out as reasons explaining the disparities in the number of clinical trials worldwide [73]. In response to this, some well-succeeded initiatives for promoting research and networking were implemented in Sub-Saharan Africa, such as the multicentric Men of African descent and Carcinoma of the Prostate (MADCaP) Consortium [74] and the African Esophageal Cancer Consortium (AfrECC) [75].

There is a need to urgently generate country-specific relevant cancer research in Mozambique. This strategy may include strengthening research capacity at the organizational, network, and policy levels for long-term sustainability.

## 9. Conclusions

The current situation in Mozambique regarding the different strategies for cancer prevention and control is worrisome. The country has several gaps to address, from an adequate cancer registration system to the creation of comprehensive cancer care centers, adequate training of personnel, and effective screening and prevention actions.

Despite the efforts being made by the government and health professionals, the country urgently needs actions to compel the various national and international entities regarding the emerging needs in the context of cancer prevention and control in Mozambique. This is paramount for creating and developing strategies to help the health sector implement a more effective and realistic national cancer control plan and to organize basic conditions for cancer care.

## Figures and Tables

**Table 1:** Cancer survival data estimates from Mozambique.

Population-Based Survival Estimates			
Author, Year	Cancer	Sample Characteristics	Survival
Joko-Fru, 2020 [26]	Breast	Patients diagnosed in 2015 with breast cancer, randomly selected from the Maputo population-based cancer registry (N = 42 ^a^)	1-year: overall survival: 75.8%; ASRS: 73.2% (95% CI: 38.0–90.5)
Sengayi-Muchengeti, 2020 [27]	Cervical	Patients diagnosed in 2015 with cervical cancer, selected from the Maputo population-based cancer registry (N = 112)	1-year: overall survival: 66.2% (95% CI 54.2–75.7); relative survival: 58.7% (95% CI 47.1–68.6) 3-year: overall survival: 55.2% (95% CI 31.6–73.6); relative survival: 54.8% (95% CI 40.2–67.2)
Gullickson, 2021 [25]	Colorectal	Patients diagnosed in 2014–2015 with colorectal cancer, randomly selected from the Maputo population-based cancer registry (N = 23 ^b^)	1-year ^c^: overall survival: 67.6%; ASRS: 82.7% (95% CI 67.0–91.4)
Hospital-Based Survival Estimates			
Author, Year	Cancer	Sample Characteristics	Survival
Come, 2018 [29]	Esophageal	Patients admitted to the MCH, diagnosed between January 2012 and December 2016 (N = 350)	Median survival time: 3.5 months for all patients; 8.7 months for patients treated with curative intent (surgery alone or with neoadjuvant chemotherapy)
Brandão, 2021 [28]	Breast	Patients with a pathological diagnosis treated in the oncological unit of the MCH, diagnosed between January 2015 and February 2016 (N = 98)	3-year overall survival: 44.8% (95% CI: 34.2–54.8) for cancer stage 0-IV and 48.0% (95% CI: 35.9–59.1) for cancer stage 0-III
		Patients with a pathological diagnosis treated in the oncological unit of the MCH, diagnosed between March 2016 and August 2017 (N = 107)	3-year overall survival: 62.6% (95% CI: 51.8–71.6) for cancer stage 0-IV and 73.0% (95% CI: 61.3–81.6) for cancer stage 0-III

ASRS, Age-standardized relative survival (International Cancer Survival Standard); CI, confidence interval; MCH, Maputo Central Hospital. ^a^ Corresponding to approximately 64% of all breast cancer cases identified by the Maputo population-based cancer registry for the period considered. ^b^ Corresponding to approximately 64% of all colorectal cancer cases identified by the Maputo population-based cancer registry for the period considered. ^c^ The results presented for the 1- and the 3-year overall survival were the same.

## Data Availability

Not applicable.
